# Hormone replacement therapy and outcomes for women with non-small-cell lung cancer: can an association be confirmed?

**DOI:** 10.3747/co.v16i3.302

**Published:** 2009-05

**Authors:** O. Ayeni, A. Robinson

**Keywords:** Sex, lung cancer, hormone therapy, prognostic factors

## Abstract

**Background:**

A recent report suggested that women who had been taking hormone replacement therapy (hrt) experienced significantly decreased survival after a lung cancer diagnosis. Given the large cohort of women who have received hrt, it is important to try to confirm that association.

**Methods:**

We reviewed female patients diagnosed with lung cancer at our institution between January 1999 and December 2003 for age at diagnosis, disease stage, treatment, smoking history, hrt, performance status, weight loss, age at menopause, and overall survival. Patients were excluded if they had small-cell lung cancer or an unknown primary cancer, or if they had had previous or synchronous non-lung, non-skin cancers. Statistical analysis used the chi-square test for categorical variables and the Kaplan–Meier method and Cox regression model for univariate and multivariate analyses of overall survival.

**Results:**

Of 397 eligible patients, most (68%) were stage iii or iv. The group included very few never-smokers (5%). The proportion of patients with experience of prior or current hrt was 29%, and no effect on overall survival was observed. Median survival was 13 months in the non-hrt group and 14 months in the hrt group. Significant factors predicting for overall survival included performance status, stage, and weight loss.

**Conclusions:**

Stage, performance status, and weight loss are the most powerful predictors of survival for women with non-small-cell lung cancer. As compared with non-hrt users, patients with prior hrt use did not have inferior outcomes, failing to duplicate previously published results.

## 1. INTRODUCTION

Sex-related differences in lung cancer outcome have garnered a significant amount of interest recently^1^. Sex-related differences in lung cancer pathology (women more often have adenocarcinoma, more women with lung cancers have K-*ras* mutations, and so on)^2^,^3^, lung cancer risk factors (women appear to have an increased risk of cancer at lower levels of tobacco exposure)^4^–^6^, natural history (women appear to have cancers with a slower doubling time)^5^,^7^, and prognosis (women appear to have better survival with advanced disease)^8^,^9^ have led to the examination of sex-specific factors that may be at work in lung cancer. Much work has focussed on hormones such as estrogen and progesterone^10^,^11^ and their effect on lung cancer development and prognosis.

Recently, a study by Ganti *et al.*^12^ suggested that the use of hormone replacement therapy [hrt (exogenous estrogen)] is associated with significantly worse outcomes among women treated for non-small-cell lung cancer (nsclc). Whether this finding was an effect of chance, confounders, a tumour-related effect, or another cancer-related effect is unclear. Although estrogen replacement therapy has not shown a consistent effect on lung cancer incidence (and may even be slightly protective^13^), the possible association of estrogen replacement therapy with an aggressive course of lung cancer was highly interesting.

Various reasons for the association documented in the paper Ganti and colleagues can be speculated upon. In addition to statistical chance, these reasons include the effects of various confounders^14^–^16^, effects of estrogen on tumour biology^7^–^19^, and effects of estrogen independent of tumour biology—for example, thrombosis risk^20^,^21^, among other possibilities.

Clearly, the importance of lung cancer as a leading cause of cancer-related death in women 22, coupled with the large magnitude of effect seen in the Ganti study, underscores the importance of trying to confirm the Ganti findings. Even though exogenous estrogen use has declined dramatically since data from the Women’s Health Initiative study were released, the issue is still very relevant 23.

## 2. PATIENTS AND METHODS

The Hôpital Régional de Sudbury Regional Hospital (hrsrh) Regional Cancer Program (rcp) is a referral centre for patients with lung cancer in northeastern Ontario. Most incident cases of lung cancer in the region (>75%) are referred to the rcp for either radiation, systemic therapy, or follow-up of early-stage disease. The present study was approved by the research ethics board of the hrsrh.

Using hrsrh-rcp records, we identified female patients who had been diagnosed with lung cancer between 1999 and 2003. Identified patients were excluded if they had small-cell lung cancer (sclc), carcinoid tumours, unknown primary tumours, or previous or synchronous non-lung, non-skin cancers and no pathology confirmation of lung cancer.

At their first clinic visit, all ambulatory patients had received a questionnaire asking for information about smoking history, weight loss, performance status, comorbid illnesses, use of hrt (current or former), previous surgeries, age at menarche and menopause, concurrent medications, and family history.

Information about stage, pathology, treatment, and outcome were extracted from the medical records, including pathology reports, radiology reports, and clinic notes. Information on hrt, smoking, and related factors was extracted from the nursing history and cross-referenced with the physician history. Use of hrt included either current or previous use of exogenous estrogen as hormone replacement. Patients using exogenous estrogen for birth control purposes were not considered to have used hrt. Patients who were on tamoxifen or raloxifene were excluded. Current smoking status included patients currently smoking or having smoked in the preceding year; past smoking included any smoking of more than 2 pack–years. For the survival analyses, cases in which death was not clearly documented were censored at the last known contact with patient.

The chi-square test was used for categorical variables, and Kaplan–Meier curves were generated for overall survival. Log-rank testing was used for survival analyses. The Cox regression model was also used for multivariate analyses.

## 3. RESULTS

We identified 397 women that fit the nsclc patient criteria and that had been diagnosed between 1999 and 2003. Table i lists the characteristics of those patients. In addition, we examined 87 patients with sclc.

Notably, only 5% of patients were never-smokers, this low proportion possibly being the result of the very high smoking rate in the population within the catchment area for the hrsrh 24. This proportion is also consistent with previously published research from the area, which shows a more than 95% smoking rate for lung cancer patients in northern Ontario^24^.

Use of hrt was documented in 29% of the patients, no hrt use in 58%, and unknown use in 11%. The remaining 2% of the patients were known to be premenopausal. The patients with unknown hrt use were principally those who were not seen in the ambulatory clinic, but as inpatients for radiation (that is, for brain metastases or with very poor performance status), and who thus did not complete a questionnaire.

**FIGURE 1:**
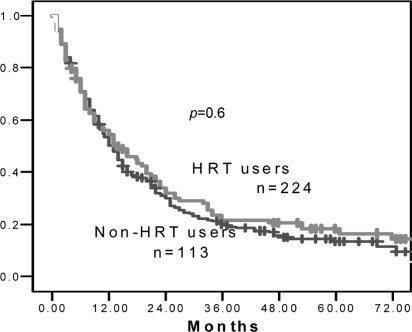
*Overall survival as a function of hormone replacement therapy* (*hrt) use. Users of* *hrt* *had a median survival of 14 months as compared with 13 months for non-users of* *hrt.*

Figure 1 shows the overall survival for women with nsclc based on whether they had or had not received hrt. No effect of hrt on overall survival was observed, median survivals being 14 months for hrt recipients and 13 months for hrt non-recipients (log-rank *p* = 0.6). The 2-year survival was also virtually identical, with 32% of patients in the hrt arm and 30% of patients in the non-hrt arm being alive at 2 years. When a multivariate analysis using stage, age, treatment type, performance status, weight loss, and use of hormone therapy was analyzed using the Cox regression model, there was still no significant association with hrt use (*p* = 0.7). In addition, given that multiple confounding treatment-related factors may have been insufficiently addressed using these models, exploratory analyses for specific subgroups of patients were attempted—for example, just patients with stage iv disease or with surgically treated stage i and ii disease. In neither of those groups was any significant trend observed. Patients with sclc were analyzed separately, and no significant difference was seen [hazard ratio (hr): 0.8 for hrt users versus non-users; median survival: 8 months for both groups].

**FIGURE 2:**
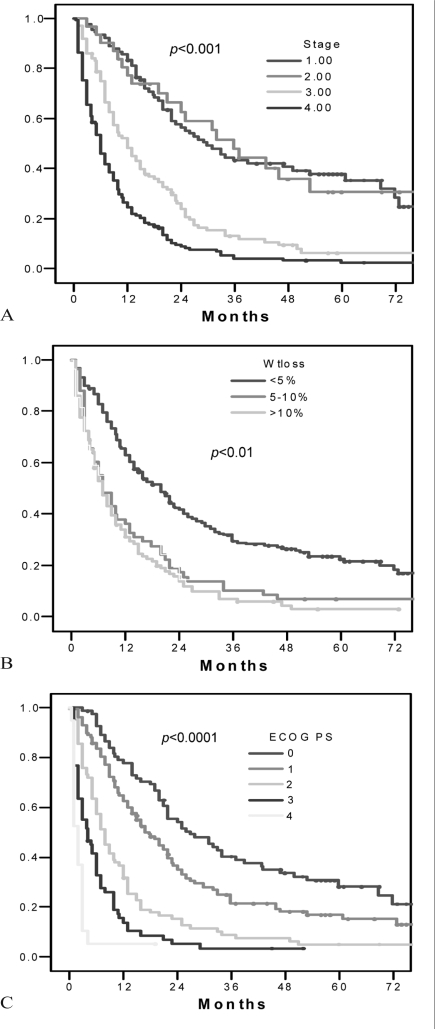
*Overall survival as a function of the commonly accepted clinical prognostic factors for non-small-cell lung cancer (nsclc), demonstrating (A) clear separation by clinical stage; (B) clear separation by weight loss; and (C) clear separation by Eastern Cooperative Oncology Group (ecog) performance status.*

In contrast, Figure 2 shows the association of survival with the known prognostic factors for lung cancer—most notably, stage, performance status, and weight loss (*p* < 0.01 for all comparisons). In multivariate analyses, stage and performance status retained their significance (both with *p* < 0.02), but weight loss did not (*p* = 0.1). In multivariate analyses using stage, age, weight loss, initial treatment, and performance status, hrt use was not associated with inferior outcomes (hr: 0.8; *p* = nonsignificant).

Looking at subgroups, the cohort included too few never-smokers (only 5% of total) to do an adequately powered subset analysis. We observed no association with estrogen replacement and survival in any subgroup based on stage, initial therapy, age, or tumour histology. Other markers of estrogen exposure, such as age of menarche and menopause, were not evaluated. Prior hysterectomy was documented for roughly 30% of the patients, making an accurate estimation of age at menopause difficult.

## 4. DISCUSSION

The previously published association between hrt use and adverse lung cancer outcomes was not confirmed using this independent data set. Commonly accepted prognostic factors for lung cancer in general—notably performance status and stage, maintained their robust association with overall survival in the women studied 24,25.

Several factors may explain the lack of association seen in our study as compared with the study by Ganti *et al.* Limitations in both studies include a lack of information on the total duration of hrt use, the indications for hrt, and whether hrt use continued after diagnosis. For instance, in the later period, the use of combined hrt was widely known to significantly increase thrombosis risk 26, and it is probable that hrt would have been stopped after a diagnosis of lung cancer for this reason. In contrast, the commentary for the Ganti study made the assumption that women using hrt who were diagnosed with lung cancer would continue their hrt use after diagnosis. It is possible that some of these unmeasured variables affected the conclusions.

A second important difference is that our study did not initially include patients with histologies other than nsclc. Although this was a difference in the studies, it is extremely unlikely that the differences in outcome can be attributed solely to this difference in methodology. Approximately 24% of patients in the study by Ganti *et al.* had sclc. Given that sclc and nsclc behave differently and that much of the previous work on the association between estrogen and lung cancer has been in the nsclc population, we elected to include only patients with nsclc histology in our primary analysis. However, even when the sclc population was looked at separately, no clear effect was observed.

A third, and probably the most important, difference between the studies is the dramatic difference in overall survival. In our study, 5-year survival was in only the 20% range for both hrt and non-hrt users. On the other hand, median survival in the Ganti study for the non-hrt users was more than 6 years, and median survival for the hrt users (18% of the total) was more than 3 years. The survival differences between that study and ours are significant, suggesting that these two populations were different, either because of referral bias or practice difference. Even a 39-month median survival for an unselected lung cancer population is impressive when compared with U.S. Surveillance, Epidemiology, and End Results (seer) database data showing a 5-year survival of roughly 15% for women with lung cancer in 1995^27^. In fact, in looking at the 47% of patients in the Ganti study who had stage iii or iv disease, even the 39-month median survival for hrt users is rather impressive. The survival data from our centre are consistent with survival data from across Ontario in lung cancer 28 and closer to the expected survival rates for women with lung cancer documented in the seer database 27.

Thus, although it is possible that hrt and estrogen play a role in the survival of women with lung cancer, the degree of difference seen in the paper by Ganti *et al.* was certainly not duplicated in our study. This difference may be the result of a select group of patients with an extremely good prognosis, but in general, other factors such as stage and performance status significantly outweigh hrt as a prognostic factor.

Clearly, the present study has all the limitations of a retrospective methodology, as did the Ganti study. No firm conclusions can be drawn regarding the role of hrt on lung cancer survival in women. Further evaluation concerning the role of estrogen and other hormones in the development, progression, and treatment of lung cancer in women is indicated. Information on estrogen interactions with *MDM2* single-nucleotide polymorphisms may be particularly intriguing. However, the present study does not support the notion that current or prior use of hrt is, in itself, an adverse prognostic feature of significance in nsclc.

## 5. CONFLICT OF INTEREST

The authors declare that no conflicts exist.

## Figures and Tables

**TABLE I:** Baseline characteristics of the study patients

*Characteristic*	*Value*
Patients (*n*)	397
Stage (%)	
i	24
ii	18
iii	26
iiv	42
Age group (%)	
<60 years	31
60–75 years	50
>75 years	19
Median age (years)	66
Smoking status (%)	
Never-smoker	5
Ex-smoker	22
Current smoker	71
ecog performance status (%)	
0–1	56
2	22
3–4	21
Weight loss (%)	
<5%	48
5%–10%	15
>10%	29
Histology (%)	
Adenocarcinoma	41
Squamous cell	40
Large cell	5
nsclc (not defined)	12
Bronchioalveolar	2
Hormone replacement (%)	
Yes	29
No	58
Unknown	11
Premenopausal	2
Initial treatment at diagnosis (%)	
Surgery alone	15
Surgery and adjuvant chemotherapy	2
Chemotherapy alone	32
Radiation alone	28
No treatment	10
Combined modality (chemorads)	10
Surgery and adjuvant radiation	2

ecog = Eastern Cooperative Oncology Group; nsclc = non-small-cell lung cancer; chemorads = chemotherapy plus radiation.
